# Materials and characterization techniques for high-temperature polymer electrolyte membrane fuel cells

**DOI:** 10.3762/bjnano.6.8

**Published:** 2015-01-07

**Authors:** Roswitha Zeis

**Affiliations:** 1 Karlsruhe Institute of Technology, Helmholtz Institute Ulm, D-89081, Ulm, Germany

**Keywords:** binder, catalysts, characterization techniques, high-temperature polymer electrolyte membrane fuel cell (HT-PEMFC), membrane electrode assembly (MEA), phosphoric acid-doped polybenzimidazole (PBI)

## Abstract

The performance of high-temperature polymer electrolyte membrane fuel cells (HT-PEMFC) is critically dependent on the selection of materials and optimization of individual components. A conventional high-temperature membrane electrode assembly (HT-MEA) primarily consists of a polybenzimidazole (PBI)-type membrane containing phosphoric acid and two gas diffusion electrodes (GDE), the anode and the cathode, attached to the two surfaces of the membrane. This review article provides a survey on the materials implemented in state-of-the-art HT-MEAs. These materials must meet extremely demanding requirements because of the severe operating conditions of HT-PEMFCs. They need to be electrochemically and thermally stable in highly acidic environment. The polymer membranes should exhibit high proton conductivity in low-hydration and even anhydrous states. Of special concern for phosphoric-acid-doped PBI-type membranes is the acid loss and management during operation. The slow oxygen reduction reaction in HT-PEMFCs remains a challenge. Phosphoric acid tends to adsorb onto the surface of the platinum catalyst and therefore hampers the reaction kinetics. Additionally, the binder material plays a key role in regulating the hydrophobicity and hydrophilicity of the catalyst layer. Subsequently, the binder controls the electrode–membrane interface that establishes the triple phase boundary between proton conductive electrolyte, electron conductive catalyst, and reactant gases. Moreover, the elevated operating temperatures promote carbon corrosion and therefore degrade the integrity of the catalyst support. These are only some examples how materials properties affect the stability and performance of HT-PEMFCs. For this reason, materials characterization techniques for HT-PEMFCs, either in situ or ex situ, are highly beneficial. Significant progress has recently been made in this field, which enables us to gain a better understanding of underlying processes occurring during fuel cell operation. Various novel tools for characterizing and diagnosing HT-PEMFCs and key components are presented in this review, including FTIR and Raman spectroscopy, confocal Raman microscopy, synchrotron X-ray imaging, X-ray microtomography, and atomic force microscopy.

## Introduction

Fuel cells are among the enabling technologies toward a safe, reliable, and sustainable energy solution. Yet, the lack of clean hydrogen sources and a sizable hydrogen infrastructure limits the fuel-cell applications today. Due to their elevated operating temperatures, between 150 and 180 °C, HT-PEMFCs can tolerate fuel contaminants such as carbon monoxide (CO) and hydrogen sulfide (H_2_S) without significant loss of performance [1–5]. These are typical byproducts of the steam reforming process, which produces hydrogen from hydrocarbon fuels such as methanol or natural gas. So it is an appealing concept to couple a HT-PEMFC stack directly with a fuel processor [6–7]. These so-called auxiliary power units (APU) use the fossil fuel resources more efficiently and help to reduce the emission of CO_2_. This might also be a good strategy for the wide deployment of fuel cells before the hydrogen infrastructure is established. The efficiency of the fuel cell system can be further increased by reusing the exhaust heat produced during electrical power generation.

The water management in low-temperature polymer electrolyte membrane fuel cells (LT-PEMFCs) operating between 60 and 100 ºC is highly complex. A lot of research effort has been devoted to this subject [8–10]. The most commonly used membrane material for this type of fuel cell is perfluorinated sulfonic acid (PFSA) polymer, which functions only in a highly hydrated state. On the other hand, water droplets which are building up underneath the gas diffusion layer (GDL) and the flow channels can partially block the gas supply of the cell. Therefore balancing the water content is a delicate task for LT-PEMFCs. In comparison, HT-PEMFCs are far more forgiving regarding the water management. Acid-based PBI-type membranes exhibit a high proton conductivity even in an anhydrous state. Therefore, additional humidification of the gas feeds is not needed. Operating above the water boiling temperature leads to further simplifications as there is only a single phase, the water vapor, present in the catalyst layer. This means that the transport of water in the membrane, electrodes and diffusion layer is easier. Consequently, the structure of the gas diffusion electrode and the design of the flow field plate have only minor effects on the cell performance [11–13] and can be greatly simplified.

The reaction kinetics should also benefit from higher operating temperatures. The exchange current density (*j*_0_) increases exponentially with temperature. However, the specific adsorption of the phosphoric acid electrolyte is known to hamper the oxygen reduction reaction activity on the cathode side. Moreover, the low solubility and diffusivity of oxygen in concentrated phosphoric acid has a negative effect on the ORR [14–15]. These problems are specific to phosphoric acid and not intrinsic to HT-PEMFCs. Alternative electrolytes such as ionic liquids or solid acids might solve the problem and accelerate the oxygen reduction reaction kinetics.

The benefits of operating the fuel cell at elevated temperatures include improved catalyst activity, higher tolerance to impurities such as carbon monoxide in the hydrogen fuel, and much simplified thermal and water management of the system. Hence, there are good reasons to develop fuel cell systems that can operate above 120 °C. At this moment, however, no commercial HT-PEMFCs have been developed to meet the reliability and cost requirements. Both academic and industrial research laboratories are working intensively to get HT-PEMFC technology ready for the market. Great progress has been made over the recent years in the field of HT-PEMFCs, which has been documented in many review articles [4,13,16–20]. The focuses of these investigations were mainly proton-conducting membranes and other materials of the HT-MEA. The techniques available for characterizing these materials were not discussed. Appropriate diagnostic tools are needed to understand the fundamental principles of fuel cell operation at elevated temperatures. Very recently, many advances have been made in this field. This came relatively late because material testing at high temperatures is generally very challenging and the presence of corrosive liquids such as phosphoric acid complicates it even further. Many standard test methods and procedures for LT-PEMFCs cannot be simply adopted for HT-PEMFCs. Adequate tools and tests must be developed to characterize HT-PEMs and their components accurately.

The first part of this review gives a brief summary of materials currently used in HT-PEMFCs. We then present an overview of advanced analytical tools, including novel imaging and spectroscopic techniques, which had been used to characterize HT-PEMFC materials either in situ or ex situ. We focus mainly on fuel cells based on phosphoric-acid-doped PBI membranes as these are the closest to commercialization. But many characterization techniques discussed here are applicable to other types of HT-PEMFCs as well.

## Review

### Materials for HT-PEMFCs

#### Phosphoric-acid-doped polybenzimidazole-type membranes

**Figure 1:**
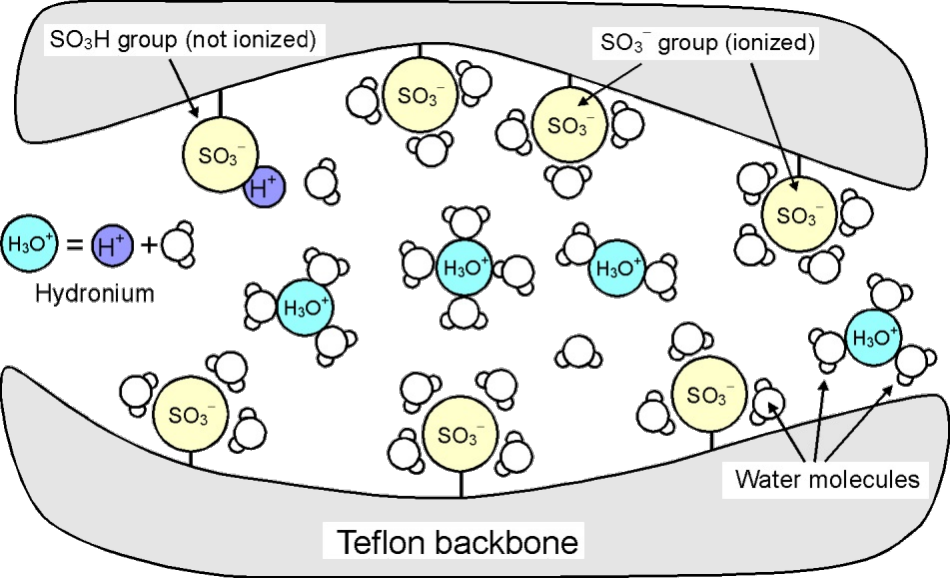
Illustration of the proton-conduction mechanisms of Nafion. Nafion polymer chains self-align into a micro channel structure. The sulfonic acid functional groups along the channel wall enable hydronium conduction.

Nafion^®^ (DuPont), the most prominent member of the PFSA membrane group, exhibits an extremly high proton conductivity of up to 0.1 S·cm^−1^ under fully hydrated conditions. This can be explained by the molecular structure of Nafion shown in Figure 1. The polytetrafluoroethylene (Teflon^®^)-like molecular backbone gives Nafion its mechanical and chemical stability, while the sulfonic acid functional groups (–SO_3_^−^H^+^) provides charge sites for proton transport. Nafion polymer chains aggregate and create voids and channels with walls covered by sulfonic acid functional groups. In the presence of water, protons (H^+^) detach from the sulfonic acid groups and combine with water molecules to form hydronium complexes (H_3_O^+^). To function properly, Nafion needs to be 100% humidified. At temperatures above 100 °C and under ambient pressure, water will evaporate instantly from the membrane. Under such conditions the Nafion membrane is a complete insulator.

**Figure 2:**
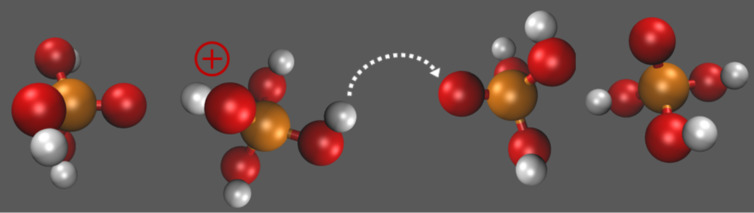
Illustration of the proton conducting mechanisms in phosphoric acid (Grotthuss mechanism). 'Excess' proton or protonic defect diffuses through the hydrogen bond network of phosphoric acid.

To avoid this problem in HT-PEMFCs, water is replaced with a less volatile liquid such as phosphoric acid (H_3_PO_4_). Phosphoric acid is thermally stable at temperatures even above 100 °C. The proton conductivity mechanism is proton hopping between H_4_PO_4_^+^ ions, H_3_PO_4_ molecules, and H_2_PO_4_^−^ ions (Figure 2). In 1995, Savinell and co-worker [21–22] proposed to use PBI impregnated with phosphoric acid as a potential electrolyte for HT-PEMs, which is still one of the most promising concepts so far. PBI (poly[2,2'-(*m*-phenylene)-5,5'-bibenzimidazole]) itself is an aromatic heterocyclic polymer. The aromatic backbone provides excellent thermal stability with a glass transition temperature of 430 °C, good chemical resistance, and high mechanical strength. To achieve adequate proton conductivity for fuel cell operation (more than 0.05 S·cm^−1^), however, PBI needs to be doped with acid because its intrinsic conductivity is very low (about 10^−12^ S·cm^−1^) [17]. During the doping process the membrane takes up a large amount of phosphoric acid. The proton conductivity of the fully doped PBI membrane at 200 °C (0.07 S·cm^−1^) [23] is almost as high as that of fully hydrated perfluorinated membranes and far less dependent on the relative humidity, thus allowing its use in HT-PEMFCs without humidifying the gas reactants.

A HT-PEMFC based on phosphoric-acid-doped polybenzimidazole membranes shares many common features with the classical phosphoric acid fuel cell (PAFC), which also utilizes phosphoric acid as the electrolyte. Unlike the electrolyte system used in a PAFC, silicon carbide (SiC) soaked in acid, the acid-doped PBI membrane is essentially solid and is therefore easier to handle. It is also more tolerant towards pressure differences between cathode and anode and the leaching of phosphoric acid from the PBI polymer during fuel cell operation is less of a concern.

**Figure 3:**
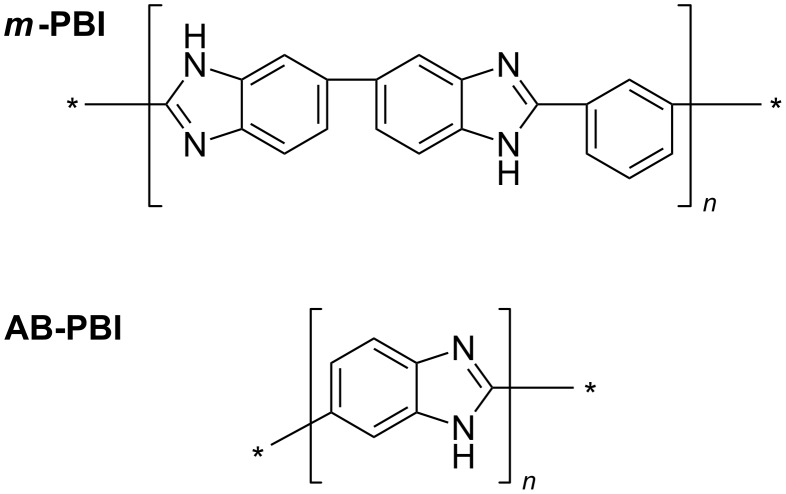
Chemical structures of *m*-PBI (poly[2,2-(*m*-phenylene)-5,5-bibenzimidazole]) and AB-PBI (poly(2,5-benzimidazole)). *m*-PBI and AB-PBI are the two most prominent members of the PBI family. These are aromatic heterocylic polymers containing benzimidazole units.

Besides PBI, there exist a great number of synthetically modified PBI polymers, which can be used as a possible host matrix material for phosphoric acid. Their properties and synthesis are described in many articles and reviews [4,17,19]. Poly(2,5-bibenzimidazole) (AB–PBI) is an important member of this class of materials. It is similar to PBI but does not have the connecting phenyl ring. The chemical structures of PBI and AB-PBI are presented in Figure 3. When fully doped, both polymer membranes show similar fuel cell performances. AB-PBI is the only commercially available membrane material for HT-PEMFCs. Membrane sheets (fumapem^®^ AM) can be ordered from FuMA-Tech GmbH, Germany. This certainly adds to its popularity and explains why its properties have been so intensively studied [12,24].

**Figure 4:**
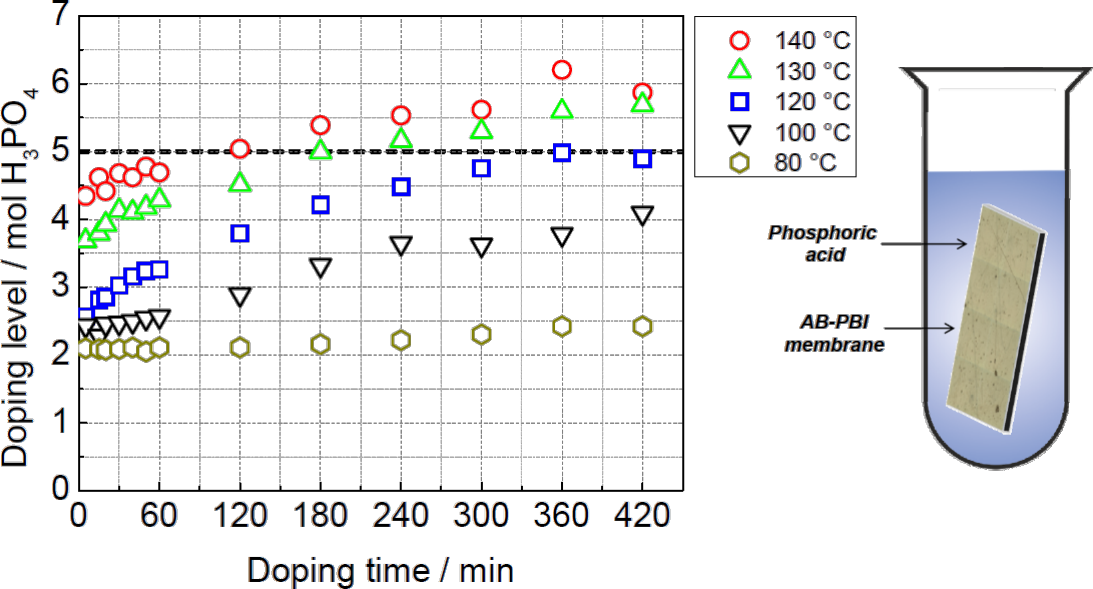
Doping process of an AB-PBI membrane in hot phosphoric acid at various temperatures. The weight increase of AB-PBI membranes correlates with the phosphoric acid solution temperature and immersion time (left). Schematic drawing of an AB-PBI membrane immersed in hot acid (right). Reprinted with permission from [34]. Copyright 2014 Elsevier.

**Doping strategies for PBI-type membranes:** The polymer matrix needs to incorporate a large volume of phosphoric acid to achieve sufficient proton conductivity. The acid doping of the membrane can be performed in various ways. One method is simply immersing the PBI-type membrane sheet into hot phosphoric acid [25]. The immersion time in the acid and the acid bath temperature defines the doping level of the membrane (Figure 4). For instance AB-PBI membranes (FuMA-Tech), doped at 120 °C, can absorb phosphoric acid up to 2.5 times their own weight, which corresponds to a chemical formula AB-PBI·5H_3_PO_4_. Notably, the acid up-take during the first few minutes in the acid is particularly large. This causes the membrane to swell considerably. The thickness doubles during the doping process, from 50 μm of the pristine material to approximately 100 μm when it is fully doped [26]. The interaction between acid and polymer host can be explained by the chemical nature of the PBI-type membranes. The polymer bears basic N-sites, which react with strong or medium-strong acids. The basic N-sites of the PBI-type polymer act as proton acceptors like in a standard acid–base reaction, and in this process ion pairs are created.

The enormous acid up-take affects the mechanical integrity of the membrane. Specifically, non-cross-linked polymers tend to dissolve in the hot phosphoric acid. For these materials another method is needed to incorporate the acid into the membrane. Wannek and co-workers [24,27] came up with a different procedure. During MEA assembly, the dry membrane sheet is attached to gas diffusion electrodes loaded with the appropriate amount of acid. The actual membrane doping process occurs then during fuel cell operation as the acid diffuses from the gas diffusion electrodes into the membrane.

Highly doped PBI membranes can also be manufactured by polymerizing the monomers directly in polyphosphoric acid. The polyphosphoric acid is then hydrolyzed to phosphoric acid, which causes a sol–gel transition of the polymer–electrolyte system creating thereby a membrane film [17]. With this method, mechanically stable membranes with acid contents of more than 95 wt % or up to 70 phosphoric acid molecules per PBI repeat unit can be manufactured [28]. It is an elegant approach to have acid doping and membrane formation in one single step.

**Figure 5:**
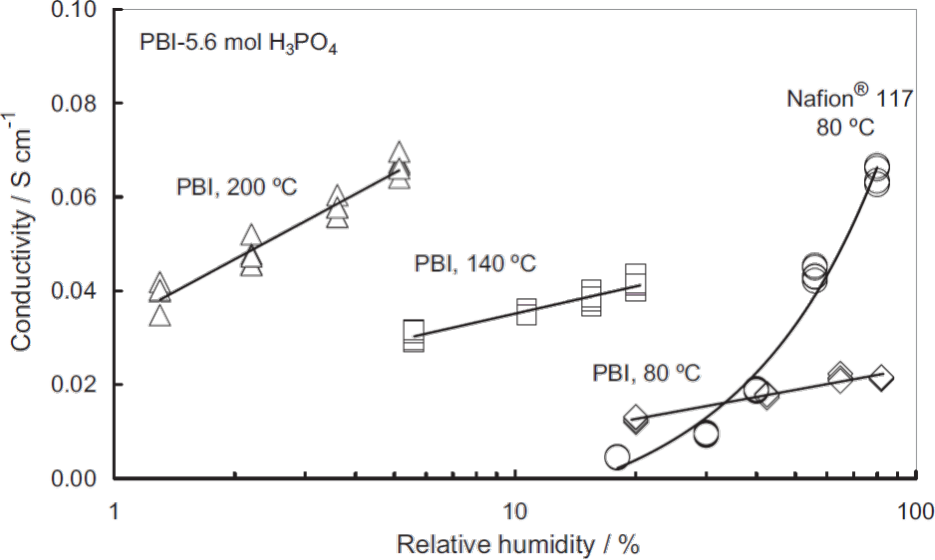
Conductivity of acid-doped PBI and Nafion as a function of humidity at various temperatures. Reproduced with permission from [23]. Copyright 2004 Wiley-VCH.

**Proton conductivity:** As soon as phosphoric acid gets in contact with the membrane material it starts to neutralize the basic sites of the polymer matrix. The PBI polymer chain has two basic nitrogen atoms per repeating unit (Figure 3) with a maximal capacity to trap two phosphoric acid molecules. Additional acid absorbed during the doping process accumulates in the free volume of the polymer chain network. It is mainly this so-call “free acid” that contributes to the proton conductivity of the membrane. The proton transport occurs through the Grotthus mechanism. Phosphoric acid has an amphoteric nature and could act as either a proton donor (acidic) or a proton acceptor (base). It forms a dynamic hydrogen bond network; in which protons can readily transfer though the formation and cleavage of covalent bonds. Figure 5 illustrates this dynamic hydrogen bond network between PBI and phosphoric acid.

**Figure 6:**
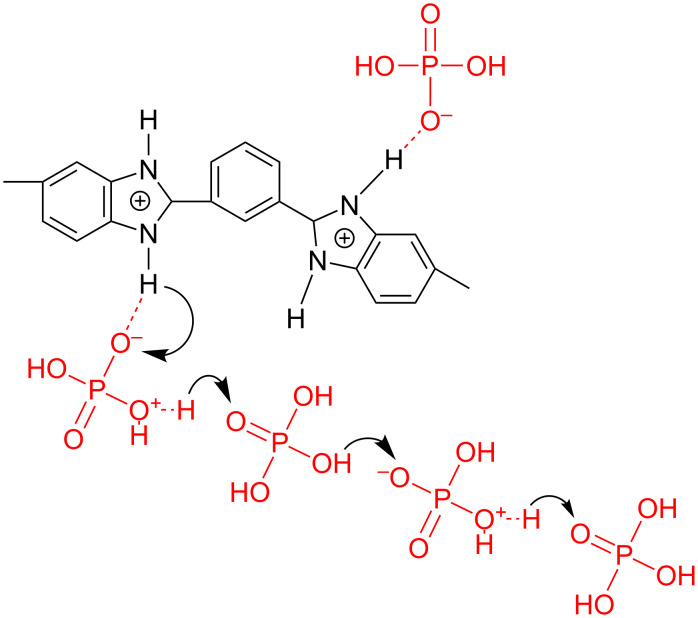
Interaction between PBI polymer host and phosphoric acid. Chemical structures of the proton transfer path along phosphoric acid molecules.

As shown in Figure 6 the conductivity of highly doped PBI is nearly as high as that of Nafion. Since the transfer of protons occurs by “hopping” though the hydrogen bond network, the conductivity of the acid-doped PBI is governed by an activation mechanism that obeys the Arrhenius law [29]. In contrast, the proton conductivity of Nafion is attributed to the vehicle mechanism, which has a weaker temperature dependence. Moreover, the conductivity of acid-doped PBI is less dependent on the relative humidity, although there is a noticeable effect.

The impact of the doping level on the conductivity has been investigated quite extensively [29–33]. At an acid-doping level lower than two acid molecules per repeating unit, proton transfer most likely happens only between protonated and non-protonated N-sites on neighboring polymer chains, resulting in very low conductivity values. The reported conductivity is approximately 10^−7^ S·cm^−1^ for dry PBI at 30 °C [29]. Increasing the temperature or the humidity level improves the conductivity only marginally. At high acid-doping levels between four and six acid molecules per repeating unit, the conductivity mechanism is similar to that of a concentrated phosphoric acid solution described in the previous section. The measured conductivity is approximately 0.07 S·cm^−1^ at 200 °C [32–33].

The conductivity depends not only on the doping level (the amount of acid) but also on the acid distribution within the membrane. For instance, AB-PBI (fumapem® AM) should remain in the hot acid bath for several hours to ensure that the acid percolates throughout the polymer network and interact with nearly every basic N-site of the polymer host. A membrane doped this way not only exhibits a higher conductivity but also retains the acid better during fuel cell operation [34]. The doping time required to achieve a uniform acid distribution depends on the properties of the polymer defined by the production process, for example, crystallinity, degree of crosslinking, and solvent residues. But this has not been investigated thoroughly.

The conductivity of PBI-type membranes is also affected by the dehydration reaction of phosphoric acid. At temperatures above 140 °C, the conductivity of phosphoric acid decreases under anhydrous conditions due to the formation of pyrophosphoric acid (H_4_P_2_O_7_), which is produced by condensing two phosphoric acid molecules and extracting a water molecule:


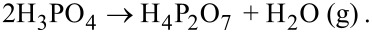


As a consequence, at 160 °C the cell resistance of PBI-type HT-PEMFC under open circuit conditions is significantly higher than with an electrical load, which helps to produce water through the fuel cell reaction. The product water rehydrates the membrane in the MEA and shifts the equilibrium between phosphoric acid and pyrophosphoric acid towards the better conducting phosphoric acid [35].

#### Gas diffusion electrodes

Conventional PEM electrodes are usually prepared by spreading a catalyst layer, a suspension of carbon-supported platinum catalyst, solvent, and binder onto a GDL, followed by a drying step. There exist a wide range of catalyst layer deposition techniques, for instance, spraying [12], decal transfer [36], painting [37], rolling [38], sputter deposition [39], and doctor blade coating [27]. Some of these methods are adapted for the fabrication of GDEs for HT-PEMFCs. In particular, spraying [12] and doctor blade coating [27] are the most common techniques used in research.

The deposition method can affect the morphology, specifically the macro structure, of the GDE significantly. For instance, the catalyst layer fabricated by doctor blade technique shows a complete network of shrinkage cracks, which go all the way though the catalyst layer. The clots are completely disconnected from each other. The situation is different for sprayed GDEs, where only small hairline cracks on the electrode surface were observed. Despite their different appearances, the sprayed and coated electrodes exhibit similar cell performances.

The absence of liquid water in the system is one of the most important differences compared with LT-PEMFCs. The distribution of the polymer binder in the electrodes and the macro structure of the electrode in general have strong effects on the performance of the LT-PEMFC cell because they are critical for water management. The fact that sprayed and coated HT-PEMFC electrodes lead to more reproducible MEA performance than typical handmade LT-PEMFC electrodes indicates the robustness of the HT-PEMFC against structural variations due to electrode preparation [12]. Still the fabrication technique might have an effect on the long-term stability of the cell. The large shrinkage cracks are the main pathway of the phosphoric acid from the membrane though the GDL and out of the cell [40]. However, to conclude this long-term tests are needed.

**Catalysts:** Similar to LT-PEMFCs and PAFCs, carbon-supported platinum is the main catalyst material used in PBI-phosphoric acid fuel cells. A difference is the noble metal loading of the GDEs, which is approximately 1 mg/cm^2^, much higher than the typical Pt loading reported for LT-PEMFCs (0.1–0.4 mg/cm^2^). The high noble-metal loading is mainly accounted to the pure utilization of platinum because the electrolyte partially floods the catalyst layer. In addition, the anion adsorption impedes the ORR in concentrated phosphoric acid. To reduce the noble-metal loading of a fuel cell, platinum alloy catalysts such as PtNi and PtCo may be used. However, the stability of these Pt alloy catalysts is questionable partially because, under fuel cell operating conditions, transition metals such as nickel and cobalt are expected to form oxides or hydroxides that tend to dissolve from the electrode surface. Nevertheless, carbon-supported platinum/transition metal alloy catalysts are often used in conventional LT-PEMFCs as well as PAFCs. There is strong experimental evidence that platinum alloys outperform pure platinum catalysts [41–44]. There are many explanations in the literature why platinum alloys exhibit better oxygen reduction reaction kinetics. In brief, the enhanced catalytic activity of platinum alloys has been credited to various structural changes of platinum caused by alloying, which may result in shortening of the interatomic Pt–Pt distance [43]. Other researchers have suggested that the alloy layer beneath the platinum skin increases the d-band vacancy of the platinum itself improving, therefore, the oxygen reduction reaction [20]. A great deal of research on this subject was carried out from 1970’s until the early 1990’s within the framework of the phosphoric acid fuel cell (PAFC) development, but there are only a few publications in the context of PBI-based HT-PEMFCs [45–46].

Rao et al. [45] prepared carbon-supported Pt–Co alloy nanoparticles of various Pt/Co atomic ratios (1:1, 2:1, 3:1 and 4:1). Theses catalysts were evaluated in HT-PEMFCs. Improved performance was observed for Pt/Co atomic ratios of 1:1 and 2:1. These HT-PEMFCs, operating at 180 °C and 50 mA/cm^2^, were stable over 50 h of fuel cell operation. Mamlouk and co-workers [46] tested commercial catalysts besides Pt/Co and also included Pt/Fe and Pt/Ni. All platinum alloys in this study had a composition of 1:1 in atomic ratio. They claimed only the Pt/Ni catalyst exhibited a better catalytic activity compared with the conventional platinum catalyst. However, both papers did not include long-term fuel cell tests so the stability of such alloy catalysts in HT-PEMs is yet to be evaluated.

**Catalyst supports:** Similar to LT-PEMFCs, high-surface-area carbon blacks (e.g., Vulcan-XC72 and Ketjen black) are often used as catalyst supports, despite the fact that operating the fuel cell under dynamic conditions (potential cycles) or at high potentials leads to severe corrosion of theses carbon materials, a drawback well-known from the PAFC research. Carbon nanotubes are a promising alternative for catalyst support because of their higher corrosion resistivity [47].

**Figure 7:**
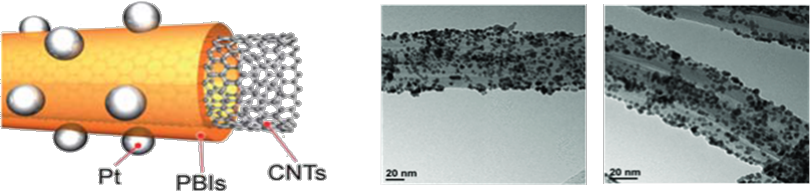
Left: Schematic drawing of an electrocatalyst composed of Pt nanoparticles loaded on the PBIs-wrapped carbon nanotubes (left). Reprinted with permission from [48], Copyright 2011 Royal Society of Chemistry. Right: TEM images of Pt nano-particles loaded on the PBIs-wrapped carbon nanotubes (right). Reprinted with permission from [49]. Copyright 2013 Wiley-VCH.

Matsumoto et al. [48] fabricated a catalyst material by wrapping individual carbon nanotubes in a PBI polymer layer covered with platinum nanoparticles. A schematic drawing and TEM images of this innovative catalyst concept are shown in Figure 7. The PBI wrapper serves as an ionomer and binder of the catalyst layer simultaneously. The polymer also glues the platinum nanoparticles onto carbon nanotubes, preventing agglomeration and detachment from the substrate. GDEs based on this type of material were prepared simply by vacuum filtration of a suspension of isopropanol and polymer-wrapped carbon nanotubes. The GDL, a carbon paper, was used as a filter. The GDEs were incorporated into MEAs for cell performances testing. The single cells achieved a peak power of over 100 mW/cm^2^ at 120 °C with a relatively low platinum loading (0.45 mg/cm^2^) for both the cathode and the anode.

**Figure 8:**
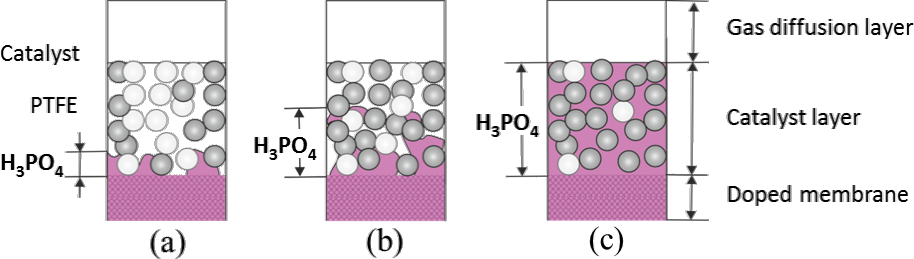
Schematic diagram illustrating the difference in the amount of phosphoric acid migration from the doped membrane to the catalyst layer with different PTFE content (a) high (b) medium and (c) low.

**Binder:** The polymer-wrapped carbon nanotube catalyst concept elegantly incorporates the binder PBI into the GDE. Similar to the selection of materials for membranes, various types of PBI can be used as binders and ionomers in the catalyst layers of HT-PEMFCs. However, PBI materials are inconvenient to handle since they are only soluble in solvents with a high boiling point [49]. Some groups managed to overcome these difficulties and incorporated PBI within the catalyst layer [48,50–51]. However, there is no clear evidence that the PBI binder actually improves the cell performance. In HT-PEMFCs, since an ion conductor (molten phosphoric acid) is already present in the GDE, an insulator such as PTFE could serve as a suitable binder [12,24]. PTFE not only glues the catalyst particles together and, hence, keeps the catalyst layer mechanically intact with its hydrophobic nature; it also controls the wettability of the GDE, which affects the infiltration of phosphoric acid into the GDE. Both the molten acid and the reactant gas need access to the active sites of the catalyst. A complete network of three phase boundaries is created, consisting of proton conducting electrolyte, electron conducting catalysts, and reactant gases. This network can be fine-tuned simply by varying the PTFE content of the catalyst layer. As shown in Figure 8, the PTFE content controls the acid migration from the doped membrane to the catalyst layer and has therefore a significant effect on the cell performance. In case of MEAs employing fumapem^®^ AM (AB-PBI·5H_3_PO_4_), the optimal PTFE content for HT-PEMFC electrodes was found to be 5% [52]. The optimum value may vary for different membrane types and doping degrees.

Nafion, the traditional binder in LT-PEMFC, is not practical for HT-PEMFCs operating at elevated temperatures. This is because under anhydrous conditions, Nafion is nonconductive and tends to encapsulate the platinum nanoparticles, resulting in blocking of the catalyst sites for hydrogen oxidation and oxygen reduction. It is even possible to prepare electrodes for high-temperature MEAs without any polymeric binder. The absence of binder material seems to affect the mechanical stability only little. MEAs built from binderless electrodes demonstrated a stable cell performance over 900 h of operation [53].

### Characterisation techniques for HT-PEMFCs

#### Optical spectroscopy

**Raman and infrared spectroscopy – Acid-doping process of PBI membranes:** Raman and infrared (IR) spectroscopy are powerful tools to study the effects of acid-doping on PBI-type polymers because they are highly sensitive to molecular structural changes that occur during the acid–base proton exchange reaction between PBI and phosphoric acid as shown in Figure 5


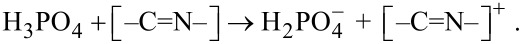


**Figure 9:**
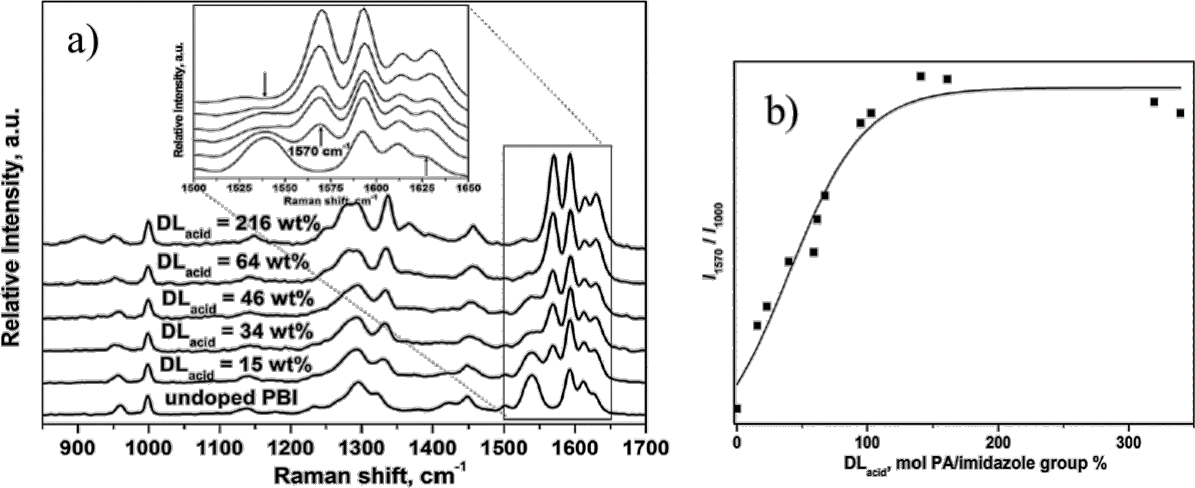
(a) Raman spectra of pristine and phosphoric acid-doped PBI. (b) Ratio of relative intensities versus acid doping level expressed in 1 M H_3_PO_4_ per polar group (%) for the peaks 1570 and 1000 cm^−1^ for PBI/ H_3_PO_4_. Reproduced with permission from [55]. Copyright 2014 Royal Society of Chemistry.

**Figure 10:**
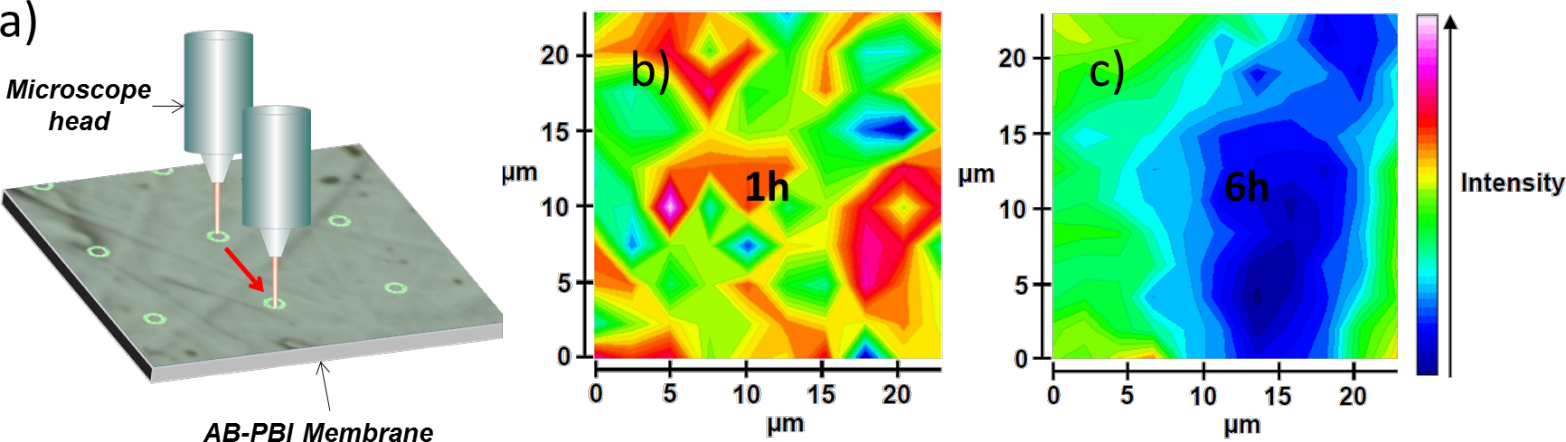
(a) Schematic drawing of confocal Raman microscopy mapping. Confocal Raman maps of phosphoric-acid-doped AB-PBI membranes. The membrane sheets were immersed in a 120 °C hot phosphoric acid bath for (b) 1 h and (c) 6 h. The Raman images show an increasing homogeneity of the phosphoric acid distribution in the membrane with prolonged doping time. Reproduced with permission from [34]. Copyright 2014 Elsevier.

They are also sensitive to molecular interactions between membrane components. Raman spectra of pristine and acid-doped PBI materials with various doping degrees have been recorded and relevant bands have been assigned [26,54–56]. Typical examples of such spectra are presented in Figure 9a [55]. The Raman band at 1000 cm^−1^ was assigned to the *meta*-benzene ring vibration, which remains roughly unchanged as the acid content in the membrane increases. In contrast, the band at 1539 cm^−1^, which is associated with the symmetric stretch of the imidazole group, becomes stronger and shifts towards 1570 cm^−1^ with increasing acid content. During acid-doping of the PBI polymer, the imidazole group becomes gradually protonated, which causes this Raman blue shift and increase of intensity. Once the protonation reaches saturation (two per repeating unit of PBI), the intensity of this band becomes constant. This is confirmed by plotting the ratio of the band intensity of 1570 cm^−1^ to that of 1000 cm^−1^ against the acid-doping level, as shown in Figure 9b [55]. The protonation of the basic N-sites does not occur homogenously within the polymer matrix. Specifically, for the commercial AB-PBI membrane material (fumapem^®^ AM), the process could take several hours until the acid reaches all areas of the membrane sheet. This slow doping process can be monitored by confocal Raman microscopy. The integrated intensities of the bands at 1570 cm^−1^ and 1611 cm^−1^ were used as indicators for the interaction between the AB-PBI host and the phosphoric acid dopant. These two peaks overlap and cannot always be resolved individually in the spectra of the ABI-PBI samples. For convenience, both were selected for creating the integrated Raman intensity maps (the peak at 1611 cm^−1^ is less sensitive to increasing doping levels). Figure 10 shows the integrated Raman intensity maps for AB-PBI membrane sheets doped for 1 and 6 h in 120 °C hot phosphoric acid. With 6 h of doping time the color distribution of the map is more uniform compare to the one of 1 h indicating that after 6 h finally all basic N-sites have been protonated throughout the whole membrane matrix [34].

**Figure 11:**
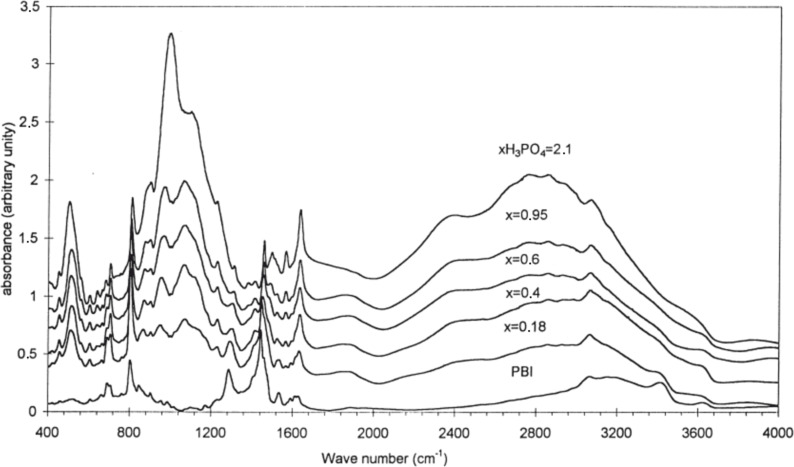
IR spectra of pristine and phosphoric-acid-doped PBI. Reprinted with permission from [29]. Copyright 1999 Elsevier.

The gradual protonation of the N-sites by transferring protons from phosphoric acid to the imidazole groups of PBI with increasing acid content can also be observed with infrared spectroscopy [29,54,57–58]. IR spectra of the pristine and acid-doped PBI films are shown in Figure 11. The most relevant region of the spectra is from 2000 to 4000 cm^−1^ since the N–H stretching modes appear in this range. According to Muso et al. [58], there are three distinguishable bands at 3415, 3145, and 3063 cm^−1^ visible in the pristine sample. The narrow peak at 3063 cm^−1^ corresponds to the stretching modes of the CH groups of the polymer backbone, whereas the other two are attributed to the various N–H stretching modes. The relatively sharp peak at 3415 cm^−1^ is assigned to the stretching vibration of isolated, non-bonded N−H groups, and the broad peak located at approximately 3145 cm^−1^ is linked to stretching vibration of self-associated, hydrogen-bonded N···H [56].

Once the PBI material is doped with phosphoric acid, a very broad absorption band appears in the wave number range between 2400 and 3000 cm^−1^, which is consistent with protonation of the N-sites on the imidazole groups of PBI. The intensity of this new band increases with the doping level, while the absorption of both the N–H groups at 3415 and the N–H∙∙∙N groups at 3145 cm^−1^ decreases [4]. The adsorption bands of the acid anion (H_2_PO_4_^−^) between 400 and 1300 cm^−1^ in the IR-spectrum can also be used as markers for the degree of protonation of PBI. Specifically, the area of the peak at 1630 cm^−1^ [29] is sensitive to the acid content in the membrane. The value increases with the doping levels until it reaches a maximum corresponding to transfer of two protons from phosphoric acid to the two imidazole groups of the repeating unit of PBI.

Di Noto and co-workers [54] investigated IR-spectra of pristine and doped AB-PBI. They also examined the lower spectral range from 400 to 2000 cm^−1^. The presence of the band at 942 cm^−1^ (attributed to H_2_PO_4_^−^ ions) suggests that when the AB-PBI is exposed to H_3_PO_4_, an acid–base proton transfer reaction occurs. As the amount of acid in the membrane increases, the AB-PBI bands are gradually masked by those of phosphoric acid. The phosphoric acid band at 998 cm^−1^ continues to grow as the amount of acid in membrane exceeds the number of imidazole sites and free phosphoric acid accumulates in the membrane. While the presence of both H_3_PO_4_ and H_2_PO_4_^−^ bands in the spectra of the acid-doped AB-PBI membranes suggests that an acid–base reaction has occurred, evidence of both these species is also present in the phosphoric acid spectrum due to the dissociation equilibrium that exists in aqueous phosphoric acid.

**IR studies of the adsorption of phosphoric acid species on platinum:** IR spectroscopy has been used as a tool to study electrochemical interfaces and to characterize adsorbed species on catalytic surfaces. Habib and Bockris [59] were the first who applied this technique to investigate the adsorption of phosphoric acid on platinum. Their goal was to determine the acid species, molecule or anion, which adsorbs onto the surface, depending on the electrode potential. The measurements were carried out in 1 M perchloric acid as the base electrolyte with various small concentrations of added phosphoric acid. They observed in their IR spectra a peak at 1074 cm^−1^ associated with a P–O stretch vibrational mode of H_3_PO_4_ molecules adsorbed on the platinum surface. The IR adsorption peak intensity varies parabolically with potential. Between 200–700 mV vs NHE the signal increases until it reaches its maximum and then decreases again. Based on this result, the authors speculated that the adsorbed species are most likely H_3_PO_4_ molecules because they are displaced by water or oxides at the platinum surface at higher potentials. This would not be the case if they were H_2_PO_4_^−^ anions. Additionally, at pH 0 only about 1% of the H_3_PO_4_ molecules dissociate into H_2_PO_4_^−^ ions, resulting in a rather small concentration of anions.

**Figure 12:**
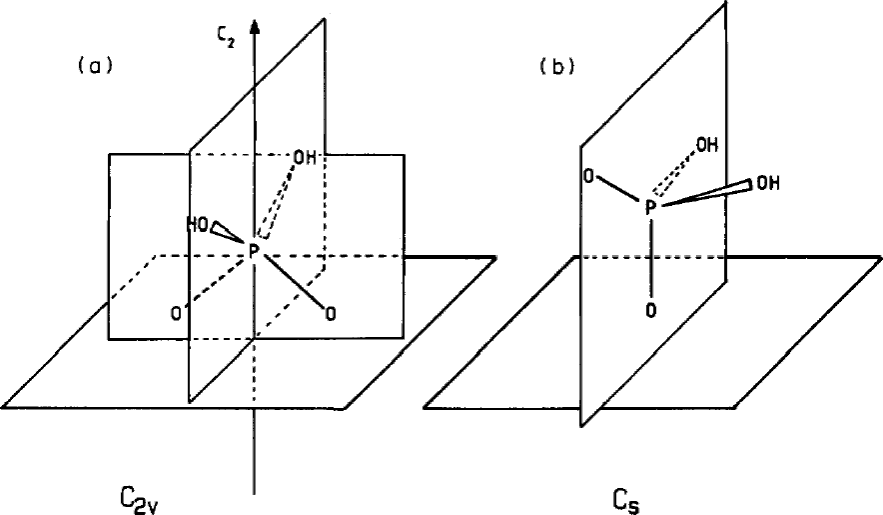
The H_2_PO_4_^−^ ion under *C*_2_*_ν_* and *C_s_* symmetries. These are the most probable orientations of H_2_PO_4_^−^ at low and high positive potentials, respectively. Reprinted with permission from [60]. Copyright 1992 Elsevier.

In 1992, Nart and Iwasita [60] conducted similar experiments and reached very different conclusions. Their FTIR instrument had much improved signal-to-noise ratio and spectral resolution. Furthermore, they measured the FTIR spectra with both s and p polarisations of light to exclude artifacts due to absorption of phosphoric acid in solution. The base electrolyte perchloric acid was replaced with hydrofluoric acid to prevent IR band interference. Nart and Iwasita found that both H_3_PO_4_ molecules and H_2_PO_4_^−^ anions could adsorb onto the platinum surface depending on the potential. They also suggested possible orientations of the adsorbates. At low potentials, the undissociated H_3_PO_4_ molecules are likely to adsorb on platinum through the non-protonated oxygen atom under the *C*_3_*_v_* symmetry. Only one IR peak at 1050 cm^−1^ associated with the P–O stretch vibration appears in the spectra. The adsorbed H_3_PO_4_ molecules are ionised to H_2_PO_4_^−^ anions as the potential increases. The onset of ionisation depends on the pH of the solution. At 900 mV, the adsorbed H_2_PO_4_^−^ anions undergo a symmetry change. Below 900 mV, the H_2_PO_4_^−^ anions adsorb to the surface through the two oxygen atoms that are not bonded to hydrogen, presenting a *C*_2_*_v_* symmetry. Above 900 mV, the dihydrogen phosphates change to a single coordination with a lower *C_s_* symmetry. The probable orientations of the H_2_PO_4_^−^ anions at different potentials are shown in Figure 12. This geometry change is most likely prompted by co-adoption of oxide spices on the platinum surface. In the IR spectra the absorption band at 1000 cm^−1^, which corresponds to the P–(OH)_2_ stretch vibration, decreases at higher potentials and indicates this symmetry change.

So far all studies had been conducted on model systems that used single crystal electrodes [61] or polycrystalline films at low electrolyte concentrations. They help us understand fundamental aspects of the interactions between phosphoric acid and the catalyst, but these model systems are far away from realistic conditions in HT-PEMFCs. The exact nature of the adsorption of phosphate species on platinum, especially in a practical fuel cell environment at elevated temperatures and much higher acid concentrations, is still not well understood. Neophytides et al. [62] constructed an electrochemical cell for in situ FTIR measurements of HT-PEMFC MEA from room temperature up to 150 °C. The MEA consisted of a phosphoric-acid-doped pyridine-based aromatic polyether membrane and platinum film electrodes. The experimental setup and cell design were validated by using CO adsorption on nanoparticles of the platinum film and electrochemical stripping. Yet there has been no published data on the issue of phosphoric adsorption from this setup.

#### X-ray absorption spectroscopy to investigate the adsorption of phosphoric acid species on platinum

Very recently, Kaserer et al. [63] published a study on the catalyst poisoning effect of H_3_PO_4_ in HT-PEM by using in operando X-ray absorption spectroscopy (XAS) incorporating the Δμ technique. The goal of this study was also to investigate phosphoric acid adsorption on platinum in a real fuel cell. The technique is capable to determine adsorbates on the platinum catalyst particles by examining the X-ray near edge structure (XANES). For these analyses, the XANES measurements were taken from −20 to 50 eV relative to the Pt L_3_ edge at 11.564 eV. The Pt L_3_ absorption edge from a platinum foil was used as a reference. The Δμ signals were generated by using the subtractive method Δμ = μ_sample_ – μ_foil_.

**Figure 13:**
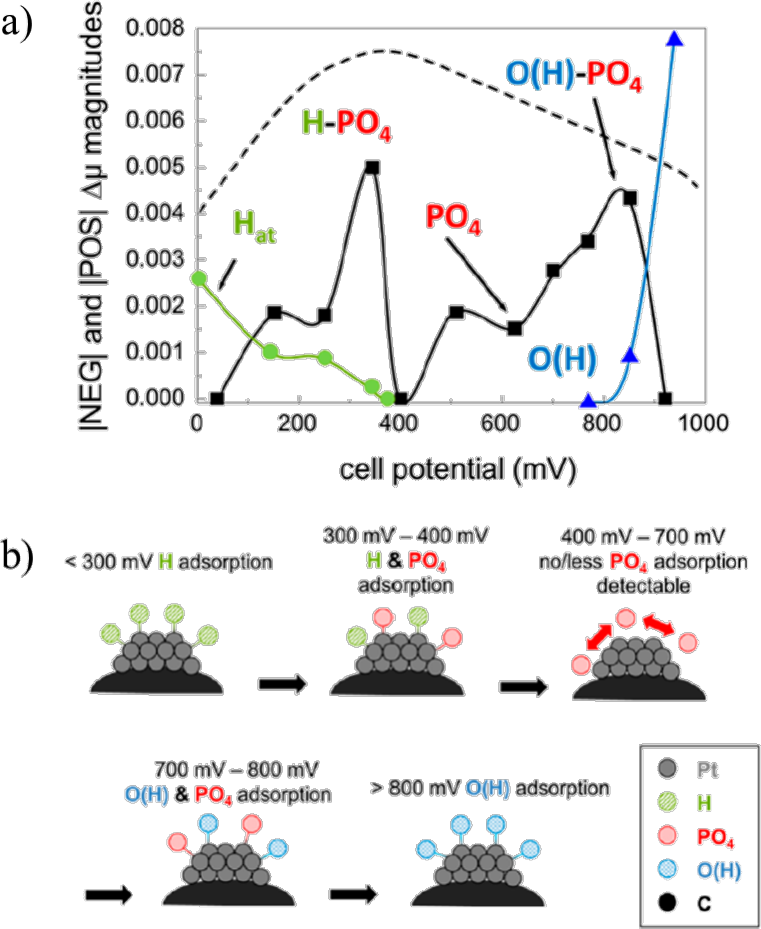
(a) Coverage of different adsorbates on platinum by analyzing |POS| and |NEG| Δμ amplitudes at various cell potentials. The dashed line indicates the expected PO_4_^3−^ coverage according to He et al. [64]. (b) Illustration of adsorbates on platinum at different cell voltages. Reproduced with permission from [63]. Copyright 2013 American Chemical Society.

With various cell potentials, different adsorbates were observed on the platinum nanoparticles as shown in Figure 13. Three potential regions were identified with distinctly different species covering the catalyst surface. At cell potentials lower than 300 mV, hydrogen is adsorbed. In the potential region between 300 and 400 mV, phosphoric acid species start to displace hydrogen and adsorb on platinum. Between 400 and 700 mV only little coverage of phosphoric acid is measured. The authors believed that the platinum surface is still fully coved with phosphoric acid in this potential range and speculated that phosphoric acid molecules or anions are very mobile on the surface and invisible to the Δμ technique. Only in the presence of other adsorbates (hydrogen and oxygen) does phosphoric acid adsorb in an ordered manner and become detectable. From 700 to 800 mV co-adsorption of oxygen and phosphoric acid species were observed. Above 900 mV only oxygen is present on the platinum surface.

These experiments provided new evidence that at higher temperatures phosphoric acid still blocks platinum atoms, thus hindering the oxygen reduction reaction. The technique, however, cannot distinguish between various adsorbing species, namely the phosphoric acid molecule or two different types of anions, on the platinum surface. Additionally, over a wide potential range (400–700 mV) the conclusion could only be drawn indirectly due to lack of measurement sensitivity.

To elucidate the origin of the reduced poisoning of Pt-alloy catalysts in the presence of phosphoric acid, Mukerjee and co-workers [64] conducted in situ X-ray absorption spectroscopy experiments. Similar to the previously described study [63], they investigated adsorbates on the catalyst surface by interpreting the X-ray near edge structure (XANES). They also applied the Δμ technique. The difference is that the measurements were carried out at room temperature while using perchloric acid as the base electrolyte with added small concentrations of phosphoric acid. Commercial carbon-supported Pt and Pt/Ni nanoparticle catalysts were used in this study. The authors concluded form their Δμ data analysis that phosphate species remain adsorbed up to a higher potential on Pt/Ni than on Pt, which prevents the adsorption of OH^−^ from the water activation. As a result more catalytic active sites are available for the oxygen reduction reaction.

The measured surface coverage of phosphoric acid on platinum as a function of the cell potential is very different from what was observed in [63]. Similar to the FTIR measurements from Iwasita et al. [60], they observed an increase of phosphoric acid adsorption starting from 0 mV. The maximum coverage in their experiments was found at a lower potential. Above 400 mV the phosphoric acid adsorption decreases. In their XANES data phosphoric acid is present on the platinum surface at all potentials, but this is not the case at elevated temperatures and higher acid concentrations as reported by Kaserer et al. [63].

#### Synchrotron X-ray radiography and tomography – acid distribution in HT-PEMFCs

Synchrotron X-ray radiography had been successfully applied to LT-PEMFCs for visualization of liquid water profiles under different operating conditions. It was also straightforward to extend the same technique to the studies of phosphoric acid concentrations and distribution changes in HT-PEMFCs [65–68]. In one of the first studies, Maier et al. [65] selected an in-plane experimental set-up to image the cross-section of an HT-PEMFC during load cycles. The cell hardware was modified to allow for a better transmission of X-rays. The attenuation coefficient of phosphoric acid is approximately 7 times higher than that of water. Hence, a beam energy of 30 keV was selected, higher than what is commonly used to visualize water in LT-PEMFCs, as a good compromise between signal intensity and selectivity to H_3_PO_4_.

**Figure 14:**
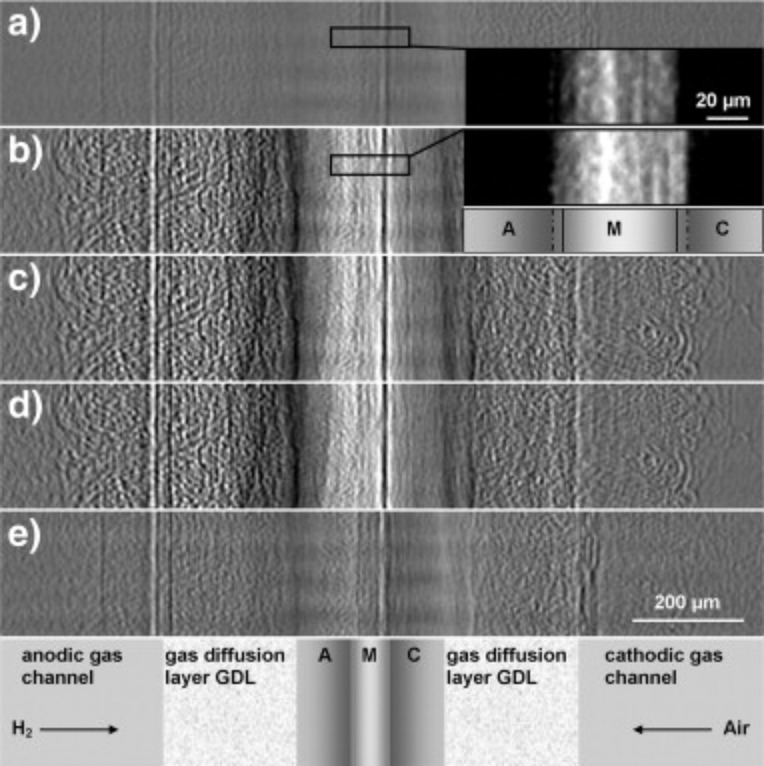
Normalized radiographs of the cross section of the MEA at different current densities *j*: a) 0 mA·cm^−2^ (OCV before), b) 140 mA·cm^−2^, c) 300 mA·cm^−2^, d) 550 mA·cm^−2^ and e) 0 mA·cm^−2^ (OCV after). Inset: Non-normalized enlarged radiographs of the membrane and parts of the catalyst layers at OCV and *j* = 140 mA·cm^−2^ (A, anode; M, membrane; C, cathode). Reprinted with permission from [65]. Copyright 2010 Elsevier.

**Figure 15:**
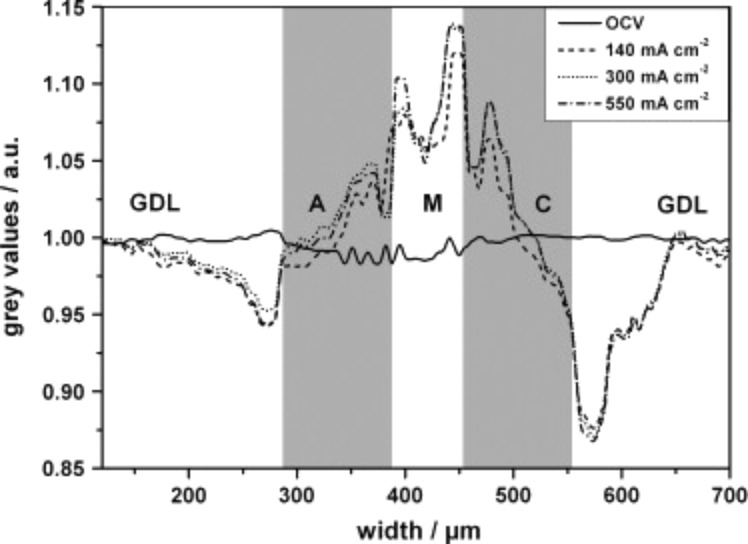
Changes of the transmission through the MEA (grey values) compared to steady-state conditions at OCV for different operating conditions: at OCV (solid line), 140 mA·cm^−2^ (dashed line), 300 mA·cm^−2^ (dotted line) and 550 mA·cm^−2^ (dot and dash line). Reprinted with permission from [65]. Copyright 2010 Elsevier.

Maier et al. [65] obtained radiographs of a HT-PEMFC during load cycle changes. The normalized radiographs of the cross-section of the MEA at different current densities are displayed in Figure 14. After changing from OCV to a current density *j* = 140 mA/cm^2^ the membrane thickness increased by approximately 20%. A further increase in current density to 300 mA/cm^2^ and 500 mA/cm^2^ did not lead to a significant additional swelling of the membrane. After returning to zero current, the membrane thickness was restored to the original value before the load cycle. The swelling of the membrane can be explained by hydration of the membrane. This is also consistent with the observation that switching the fuel cell from OCV to a current density of *j* = 140 mA/cm^2^ increases the transmission in the membrane. The changes of transmission (“grey value” in the X-ray radiograph) though the MEA are shown in Figure 15. The water production in the cell also increases the transmission of both anode and cathode catalyst layers. While the pores of the GDL get filled with hydrated phosphoric acid the transmission there decreases.

Recently, the same group reported [67] in situ X-ray radiography in the through-plane viewing direction, which allows to visualize structural changes of the electrodes during load cycles. For this study, the electrodes were prepared by coating and the catalyst layer exhibited a network of shrinkage cracks. The individual clots were clearly visible in the radiographs. During operation the membrane swelled, driving phosphoric acid and product water into the catalyst layer. This process was monitored by the change in the local transmittance of the cell during load cycles. The effect was more pronounced beneath the channel area because of the lower local compression rate of the MEA, which permitted stronger membrane deformation. The redistribution of phosphoric acid caused structural changes of the catalyst layer in the channel area. It was found that part of the electrode structure was displaced irreversibly after cell operation at load conditions that might lead to structural aging of the electrodes.

The simultaneous changes in the MEA structure and the phosphoric acid concentration make the quantitative analysis of X-ray radiographs challenging [40]. With additional 3D information, X-ray tomography is more effective in the localization and quantification of the acid electrolyte within the GDL and catalyst layer. It is necessary to establish a grayscale value reference for comparing the tomogram with known phosphoric acid concentrations. An important outcome of this research is a better understanding of acid loss during operation. Initial results indicate that the cracks in the catalyst layer and the micro porous layer (MPL) form the main pathway for phosphoric acid to escape from the interior of the MEA to the adjoining flow field.

**X-ray tomography – morphologies of MEAs and GDEs:** 3D X-ray tomography instruments are now commercially available. They are highly popular for studying internal structures of complex material assemblies. Fuel cell components such as MEAs and GDEs are certainly of such nature. Diedrichs et al. [69] used this analytical tool to investigate the impact of mechanical compression though the flow field of the bipolar plates on high-temperature MEAs. A sample holder was constructed with a serpentine flow field contact area for which the contact pressure could be adjusted between 0.5 and 2.5 MPa. From the X-ray tomography cross section images the changing thickness of the MEA was determined with increasing compression. The MEA bent itself into the flow field cannels, and bulges could be clearly seen in the X-ray tomography images. Beneath the channel area the MEA thickness increased with higher contact pressure, while the opposite occurred beneath the land area. Further compression led to irreversible structural damages of the MEA. The evaluated MEA manufactured by BASF utilized carbon paper as the GDL material. The rigid carbon fiber penetrated the soft membrane material, which resulted in small pinholes. Scanning electron microscopy (SEM) images showed broken GDL fibers piercing into the membrane and confirmed the observation made by X-ray tomography.

**Figure 16:**
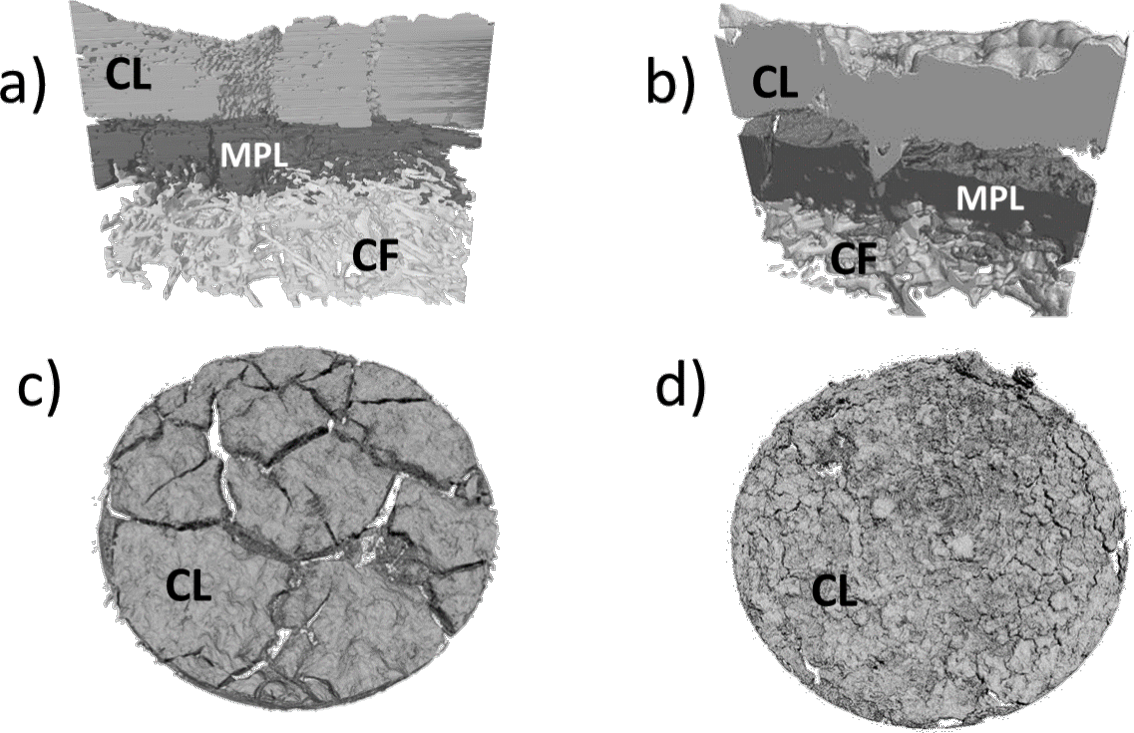
3D rendering of the X-ray microtomography data for the electrode cross section (a) coated electrode (b) sprayed electrode. 3D rendering of the X-ray microtomography data for the electrode surfaces (c) coated electrode (d) sprayed electrode. CL, MPL, and CF stand for catalyst layer, microporous layer, and carbon felt, respectively. Reprinted with permission from [12]. Copyright 2014 Elsevier.

The same analytical tool has also been used to study the morphology of various GDEs [12] with the catalyst layers either sprayed or coated onto the GDLs. The impact of fabrication techniques on the macrostructure of the GDEs is presented in Figure 16.

The coated catalyst layer showed a complete network of shrinkage cracks. The cracks went all the way though the catalyst layer. The clots were completely disconnected from each other. The scenario was quite different for the sprayed electrodes on a rough surface, where fine hairline cracks were present. Compared with the coated catalyst layer with its straight segments, the sprayed one appeared much more heterogeneous, e.g., it contained voids and void clusters of different shapes and sizes. The catalyst layer was partially delaminated from the MPL due to the fact that the solvent of the suspension drops on the surface had quickly evaporated after less than one minute for each layer. The 3D rendered image of the sprayed GDE in Figure 16 shows that the catalyst ink penetrated the MPL and filled up an exemplary large crack of MPL.

#### Neutron radiography – acid distribution in HT-PEMFCs

A substantial advantage of neutron imaging over X-rays is that there are no constraints to cell hardware due to the high transmission of neutrons though end- and bipolar plates, which are usually made of stainless steel and graphite. The attenuation of the neutrons originates mainly from hydrogen and hydrogenous compounds such as phosphoric acid. The first neutron radiography study of the acid distribution in HT-PEMFCs was published recently [70]. One of the challenges of this technique is a suitable reference for separating the attenuation due to phosphoric acid from contributions from other cell components. The authors [70] addressed this issue with isotope exchange between ^1^H (protium) and ^2^H (deuterium, D), a technique used earlier to study water transport in LT-PEMFCs. The deuteration and reprotonation of the phosphoric acid was achieved by supplying the cell with gases that were humidified with heavy water and light water, respectively. The process could be performed in less than 20 min.

The authors captured neutron radiographs of non-operating cells in both though-pane and in-plane imaging directions. The in-plane images showed that acid was present in both GDLs and, to some extent, in the flow channels and manifolds as well. The acid appeared to accumulate between the fiber bundles of the carbon cloth. To validate the technique, the authors also quantified the amount of acid of the cell using both viewing directions. Despite some minor discrepancy between though-pane and in-plane imaging directions, the quantitative analysis agreed quite well with the actual amount of acid in the cell.

#### Atomic force microscopy (conductive mode) – PTFE distribution and content of the catalyst layer

Conductive atomic force microscopy (AFM) can probe the local conductivity of a sample surface. Mack et al. [12] used this technique to measure the surface conductivity of HT-PEMFC electrodes by applying a constant potential between the AFM tip and the sample.

**Figure 17:**
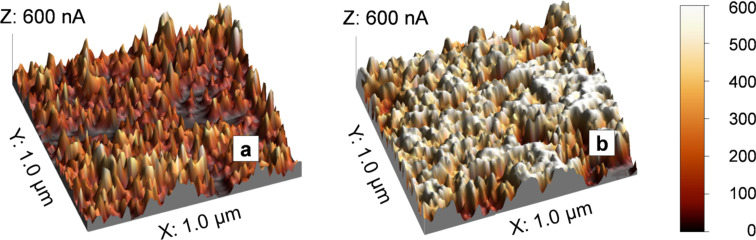
Locally resolved current maps of (a) coated and (b) sprayed gas diffusion electrode measured with the conductive AFM. The scan area is 1 μm × 1 μm. Reprinted with permission from [12]. Copyright 2014 Elsevier.

The spatially resolved current distribution maps of sprayed and coated GDE surfaces are presented in Figure 17. The bright regions of the map with high surface currents represent catalyst rich areas, whereas darker regions represent PTFE agglomerates and gas pores with much lower conductivities. The average surface conductivity of the sprayed electrode is higher than that of the coated one. The PTFE distribution within the electrode is sensitive to its preparation technique. During spraying the small droplets of the catalyst ink dried instantly on the GDL, which resulted in a homogeneous PTFE distribution in the catalyst layer. In contrast, the drying process of the coated electrode took up to 12 h, during which the light PTFE particles could migrate to the top region of the catalyst layer and form a nonconductive “skin” on the electrode surface. This PTFE-rich layer affects not only the surface conductivity but also the wettability of the catalyst layer. The high PTFE content created a hydrophobic electrode surface, which slowed down the phosphoric acid uptake during the start-up period of the MEA.

Mack et al. [52] also evaluated the surface conductivity of catalyst layers with 10% and 40% PTFE contents. Less PTFE content in the electrode led to an improved surface conductivity due to fewer PTFE agglomerates in the surface layer. The hydrophobic nature of the PTFE binder controls the phosphoric acid wettability of the GDE and, in particular, the triple phase boundary between gas reactant, electrolyte and catalyst. Atomic force microscopy was proven suitable for investigating the PTFE distribution in the catalyst layer, which has a profound effect on the start-up and steady state performance of the cell.

## Conclusion

Phosphoric acid-doped PBI-type fuel cells are so far the most promising candidates for practical high-temperature operation under ambient pressure. For commercial deployment, however, the performance and long-term stability of the high-temperature MEAs still need significant improvement. To achieve this goal, the MEA developers will rely on suitable analytical tools to evaluate single cells and their components. Great progress has been made in recent years. A broad range of characterization techniques are now available for the development of low-temperature PEM fuel cells. This has been the decisive factor behind the success of low-temperature MEA development thanks to a much better understanding of the underlying processes occurring in the MEA during fuel cell operation. We anticipate a similar trend in HT-PEMFC development. Advanced analytical tools suitable for HT-PEMFC will help optimize the MEA design and select the appropriate component materials that will withstand the harsh conditions of high-temperature operations.
